# Sialic Acid–Binding Protein-1 (SABP1) of *Toxoplasma gondii*: Preliminary Computer-Based Epitope Mapping for Enhanced Vaccine Design

**DOI:** 10.1155/japr/9909421

**Published:** 2025-09-03

**Authors:** Sarah Gholami, Ali Jebeli Eshrat Abadi, Shadan Ghiabi, Davood Siamian, Hamed Abdollahi, Farane Famili, Ali Yousefi, Hamidreza Majidiani

**Affiliations:** ^1^Young Researcher and Elite Club, Islamic Azad University, Babol, Iran; ^2^Department of Pathobiology, Faculty of Veterinary Medicine, Islamic Azad University, Babol, Iran; ^3^Department of Pathobiology, Faculty of Veterinary Medicine, Science and Research Branch, Islamic Azad University, Tehran, Iran; ^4^Department of Biology, Faculty of Basic Science, Islamic Azad University, Tonekabon, Mazandaran, Iran; ^5^Department of Parasitology, School of Medicine, Ahvaz Jundishapur University of Medical Sciences, Ahvaz, Khuzestan, Iran; ^6^Department of Biology, Faculty of Sciences, Islamic Azad University, Tehran, Iran; ^7^Students Research Center, Hamadan University of Medical Sciences, Hamadan, Iran; ^8^Department of Medical Parasitology and Mycology, School of Medicine, Hamadan University of Medical Sciences, Hamadan, Iran; ^9^Department of Basic Medical Sciences, Faculty of Medicine, Neyshabur University of Medical Sciences, Neyshabur, Iran

**Keywords:** immunoinformatics, SABP1, *Toxoplasma*, toxoplasmosis, vaccines

## Abstract

*Toxoplasma gondii* (*T. gondii*) infects one third of the human population globally, presenting serious consequences especially in pregnant women or immunosuppressed patients. This study characterized *T. gondii* sialic acid–binding protein-1 (SABP1) to determine its physicochemical, antigenic, and structural properties as well as immunogenic epitopes using bioinformatics predictions. The amino acid sequence for *T. gondii* SABP1 was analyzed using ProtParam (physicochemical properties), VaxiJen v2.0 (antigenicity prediction), AllergenFP v1.0 and AllerTOP v2.0 (allergenicity prediction), NetSurfP-6.0 (secondary structure), Robetta (tertiary structure), IEDB, IFNepitope, and IL4pred (immunogenic epitopes). The subcellular prediction was made using signal peptide, transmembrane domain, posttranslational modifications (PTMs) and protein localization). The SABP1 protein (315 residues; 33.73 kDa) possessed antigenicity (0.46), high solubility (0.783), hydrophilicity (GRAVY: −0.335), and an aliphatic index of 69.33. It was shown to be nonallergen. SABP1 is located in the cytoplasm and has no signal peptide or transmembrane domain. Importantly, there were many B- and T-cell epitopes predicted to be immunogenic, which could be beneficial for designing multiepitope vaccines to prevent *T. gondii* infection. Further validation of these epitopes using wet experiments is needed.

## 1. Introduction

The ubiquitous, abortifacient apicomplexan protozoan, *Toxoplasma gondii* (*T. gondii*), affects humans and animals worldwide, imposing socioeconomic, health, and financial burdens [1]. Given the parasite's widespread prevalence and the lack of treatments for chronic infections, vaccination remains a critical strategy to prevent *T. gondii* infection in definitive (cats) and major intermediate hosts (humans, livestock) [2, 3]. Beyond traditional approaches, a growing trend involves developing vaccine candidates using strictly screened protein fragments (i.e., epitopes) with high antigenicity and immunogenicity via in silico methods, enabling multiepitope vaccine (MEV) construction [4–8]. Although *T. gondii*'s three main secretory proteins—microneme proteins (MICs), rhoptry proteins (ROPs), and dense granule antigens (GRAs)—have been primary focuses in vaccinology (reviewed in [9–11]), many other proteins may serve as virulence factors and vaccine candidates [12]. Sialic acids (SAs) represent a diverse group of monosaccharide derivatives composed of nine carbon atoms. These SA molecules, found on glycolipid/glycoprotein surfaces, act as attachment sites for influenza viruses and enhance macrophage engulfment of *Trypanosoma cruzi* [13, 14]. SA also serves as a receptor for *Plasmodium falciparum* merozoites to recognize and invade host erythrocytes via erythrocyte-binding antigen-175 (EBA-175) interactions [15]. In *T. gondii*, micronemal proteins like TgMIC1 and TgMIC13 are specific SA binders [16, 17]. Recent studies show that SA-binding protein-1 (SABP1), located on *Toxoplasma* tachyzoite surfaces, exhibits strong SA affinity in vitro, disrupted by neuraminidase during mammalian cell binding [18]. Xing et al. demonstrated that SABP1 gene elimination renders *T. gondii* unable to adhere to or invade target cells, resulting in a nonpathogenic phenotype in mice [18]. Immunoinformatics—computer-based methods for protein analysis and immunodominant region identification—has enabled time- and cost-effective MEV design over recent decades [19]. This approach inspired our in silico study of *T. gondii* SABP1 to characterize its physicochemical/structural parameters and immunogenic epitopes. Thus, this study was an integrated immunoinformatics approach to analyze T. gondii SABP1 with the principal objective of the identification of immunodominant epitopes for the development of a MEV. Specifically, we aimed the following: (1) analyze the physicochemical, antigenic, and structural properties of SABP1 systematically, to assess its feasibility as a vaccine target; (2) identify linear and conformational B-cell epitopes, using consensus approaches afforded by multiple prediction servers; (3) predict cytotoxic T-lymphocyte (CTL) and helper T-lymphocyte (HTL) epitopes with the greatest binding affinity to human and murine MHC alleles; (4) screen epitopes based on a variety of important vaccine criteria, including antigenicity (> 0.5 VaxiJen score), nonallergenicity, solubility, nontoxicity, and potential for cytokine induction; and (5) assess epitope immunogenicity through in silico immune simulation. Ultimately, we aimed to identify an epitope priority map, such that this integrated computational assessment could be directly related to vaccine synthesis, providing viable candidates for experimental testing in the next generation vaccines for toxoplasmosis based on SABP1-mediated invasion of host cells.

## 2. Methods

Web addresses for all online servers used in this study are provided in Table 1.

### 2.1. Retrieval of SABP1 Sequence

We retrieved the SABP1 protein sequence in FASTA format from the ToxoDB server using accession number TGME49_225940 [20].

### 2.2. Physicochemical Composition Prediction

Key physicochemical properties of SABP1 were predicted using the ExPASy ProtParam server. These included: charged residue composition, isoelectric point (pI), molecular weight (MW), estimated half-life, aliphatic index, instability index, and grand average of hydropathicity (GRAVY) [21, 22].

### 2.3. Allergenicity, Antigenicity, and Solubility Profile

SABP1 allergenicity was predicted using a multimethod approach. Allergenicity predictions were performed using the AllerTOP v2.0 server (reported accuracy: 85.3%) and the AllergenFP v1.0 server (reported accuracy: 88.95%) [23, 24]. Antigenicity was predicted using the VaxiJen v2.0 server, employing a threshold of 0.45 [25, 26]. Protein solubility was evaluated using the Protein-Sol web tool. According to this tool, solubility values exceeding 0.45 indicate good solubility [27].

### 2.4. Prediction of Subcellular Localization, Posttranslational Modification (PTM) Sites, Signal Peptide, and Transmembrane Domains

Subcellular localization, transmembrane domains, and signal peptides were predicted using DeepLoc 2.0, DeepTMHMM, and SignalP-6.0, respectively [28, 29]. PTM sites were predicted using specific tools: phosphorylation sites with NetPhos 3.1, O-glycosylation sites with NetOGlyc 4.0, N-glycosylation sites with NetNGlyc 1.0, and lysine acetylation sites with GPS-PAIL 2.0.

### 2.5. Structural Evaluations and Tertiary Model Validation

Structural analysis began with secondary structure prediction using the NetSurfP-3.0 server. This tool calculated the proportions of alpha helices, extended strands (beta sheets), and random coils [30]. Subsequently, 3D models were generated using the Robetta server in fully automated mode. Robetta assigns each model a confidence score (C-score) ranging from 0 to 1, with higher scores indicating greater model reliability. The resulting 3D model was refined for structural relaxation using GalaxyRefine. Validation was then performed using the PROCHECK tool, which assesses model quality via Ramachandran plot analysis.“ (Splits sentence, clarifies “structural relaxation.

### 2.6. Continuous and Conformational B-Cell Epitopes

Linear B-cell epitopes were predicted using a multi-method approach comprising ABCpred, SVMTriP (using a fixed 16-mer length), and the ElliPro tool (handling variable lengths) [31, 32]. We selected predicted fragments common to at least two servers and further screened them for antigenicity (VaxiJen v1.0), allergenicity (AllergenFP v1.0), and solubility (PepCalc). Conformational B-cell epitopes were predicted using the ElliPro tool (part of the IEDB server) with its default parameters: a minimum score threshold of 0.5 and a maximum distance threshold of in Å [33, 34].

### 2.7. Prediction of Human and Mouse Major Histocompatibility Complex (MHC) Binders

Human MHC–binding peptides were predicted using the HLA allele reference set within the IEDB server's MHC-I and MHC-II prediction tools, following predefined recommended methods [35, 36]. For each protein sequence, the Top 10 allele-epitope combinations (based on the lowest percentile rank, indicating highest binding affinity) were selected. These represented CTL epitopes (9- or 10-mers binding MHC-I) and HTL epitopes (15-mers binding MHC-II). CTL epitopes were subsequently screened for immunogenicity (using the IEDB immunogenicity tool), allergenicity (AllergenFP v1.0), and toxicity (ToxinPred). HTL epitopes were additionally screened for antigenicity, allergenicity, toxicity (ToxinPred), and potential to induce IFN-*γ* (predicted by IFNepitope) and IL-4 (predicted by IL4pred). Similarly, the top five epitopes (per allele) binding mouse MHC-I alleles (H2-Db, H2-Dd, H2-Kb) and MHC-II alleles (H2-IAb, H2-IAd, and H2-IEd) were predicted for SABP1 using the same approach.

### 2.8. In Silico Immune Simulation

An in silico immune simulation was performed using the C-ImmSim server. This simulation models the immune response to an antigen based solely on its amino acid sequence [37]. This server models both humoral and cellular immune responses to an antigen using a position-specific scoring matrix (PSSM). The immune response to SABP1 was simulated using C-ImmSim with its default parameters.

## 3. Results

### 3.1. General Characteristics of *T. gondii* SABP1

The amino acid length of SABP1 was 315 amino acid residues. The protein has a MW of 33.73 kDa, an acidic pI of 4.98, and an in vitro half-life exceeding 30 h in mammalian reticulocytes. The protein exhibits instability (instability index = 52.10 > 40) under experimental conditions. Conversely, its aliphatic index (69.33), which correlates with thermotolerance, is relatively high. SABP1 displays hydrophilic properties, evidenced by its negative GRAVY score (−0.335). A good antigenicity score was predicted using the VaxiJen server (score: 0.46). A multimethod approach (AlgPred 2 and AllerTOP v2.0) predicted SABP1 to be non-allergenic. Moreover, the solubility score of this protein predicted by the Protein-Sol web tool was high (> 0.45), showing a score of 0.783.

### 3.2. PTM Sites, Localization, Signal Peptide, and Transmembrane Domain

**Figure 1:**
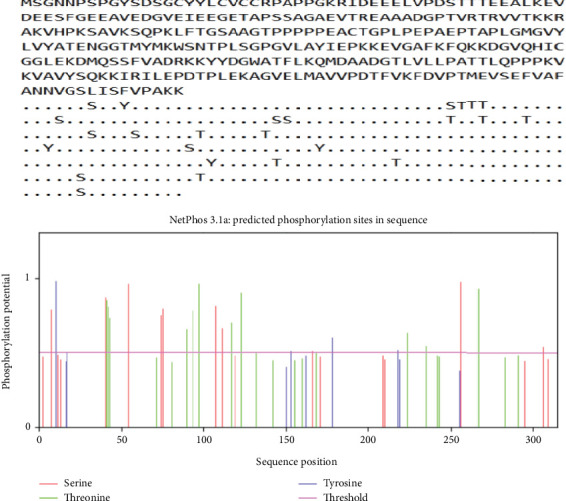
Prediction of phosphorylation sites in *T. gondii* SABP1 protein using NetPhos 3.1, showing 25 phosphorylated areas, including serine phosphorylation = 10, threonine phosphorylation = 4, and tyrosine phosphorylation = 11.

**Figure 2:**
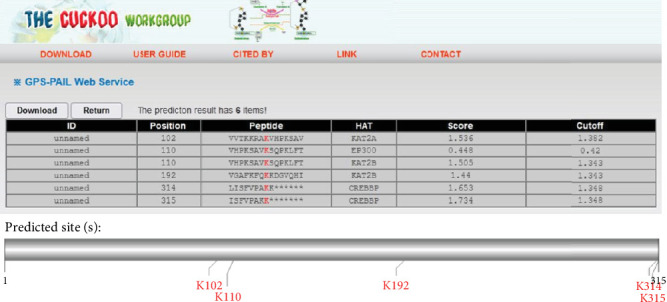
Lysine acetylation sites predicted using the GPS-PAIL web server.

No transmembrane domain or signal peptide was predicted for this protein. DeepLoc predicted cytoplasmic localization as most probable for *T. gondii* SABP1 protein (score: 0.5498). A total number of 46 PTM sites were found in SABP1 protein, mostly dominated by phosphorylation regions (serine = 10, threonine = 4, and tyrosine = 11), O-glycosylation sites (*n* = 15), and 6 lysine acetylation sites. Notably, N-glycosylation sites were absent in SABP1 (Figures 1 and 2).

### 3.3. Structural Predictions

**Figure 3:**
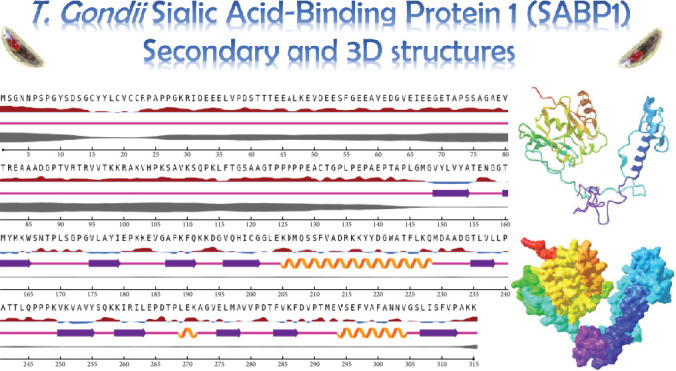
Secondary and tertiary structure prediction of SABP1 protein using NetSurfP-6.0 and Robetta web servers, respectively. Random coils and disordered regions were predominant in secondary structure, and the confidence score of the 3D model was 0.60.

**Figure 4:**
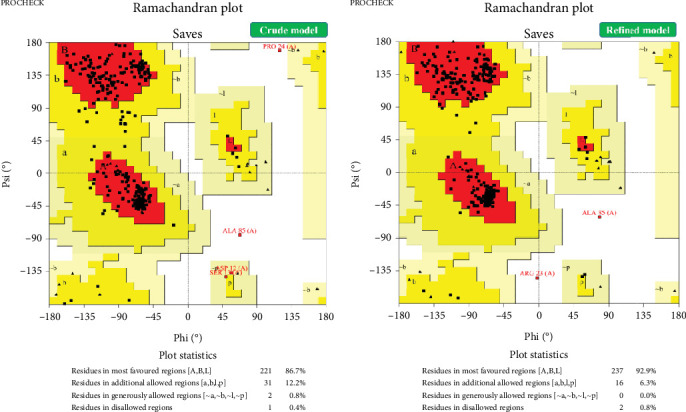
Ramachandran plot analysis estimated that about 92.9% and 6.3% of the residues in the refined SABP1 model were allocated to the most favored and additional allowed regions, respectively, showing significant enhancements in comparison to the crude model.

NetSurfP-6.0 predicted random coils as the predominant secondary structure, followed by extended strands. Approximately 50% of the structure is disordered, primarily within Residues 1–145. We predicted SABP1's 3D structure using Robetta (Baker Lab). Among five generated models, Model #1 was selected as optimal based on its confidence score (0.60; Figure 3). Model #5 emerged as the best refined structure with these validation metrics: GDT-HA (0.9817), RMSD (0.317 Å), MolProbity score (1.672), clash score (9.8), poor rotamers (0.4%), and Ramachandran-favored residues (97.1%). Ramachandran analysis revealed structural improvement in the refined model versus the initial model: most favored regions (92.9% vs. 86.7%), additional allowed (6.3% vs. 12.2%), generously allowed (0.0% vs. 0.8%), and disallowed regions (0.8% vs. 0.4%) (Figure 4).

### 3.4. B-Cell Epitope Predictions

**Figure 5:**
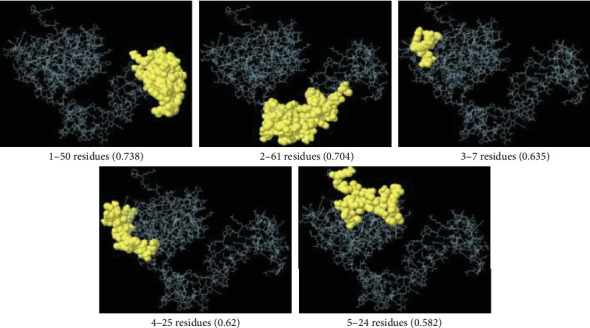
Conformational B-cell epitopes (with residue length and scores) predicted for *T. gondii* SABP1 using the ElliPro tool of the IEDB web server.

Analysis using ABCpred, SVMTriP, and ElliPro identified 20 linear B-cell epitopes common to at least two servers. Further screening in terms of antigenicity, allergenicity, and water solubility finally led to the selection of four continuous B-cell epitopic fragments as potent immunodominant regions, including: “PGKRIDEEELVPDS” (antigenicity: 1.0986), “GLEKDMQSSFVADRK” (antigenicity: 0.6031), “TREAAADGPTVRTRVV” (antigenicity: 0.7968), and “EELVPDS” (antigenicity: 1.4457) (Table 2). Based on the ElliPro server outputs, five conformational B-cell epitopes were predicted for SABP1. Details of conformational B-cell epitopes are provided in Figure 5.

### 3.5. T-Cell-Associated MHC–Binding Epitope Predictions

We predicted human MHC-I and MHC-II binding epitopes using the recommended IEDB approach with its HLA reference allele set. The Top 20 binding epitopes per MHC class, selected based on high affinity (lowest percentile rank), were subsequently screened. All 20 predicted human CTL epitopes were nontoxic; however, 12 were allergenic. Of note, only 8 out of 20 predicted human CTL epitopes demonstrated positive immunogenicity, comprising “KYYDGWATF,” “KKYYDGWATF,” “AVVPDTFVK,” “EVSEFVAFA,” “TVRTRVVTK,” “SLISFVPAK,” “EAAADGPTVR,” and “LSGPGVLAY” (Table 3). Also, 30 mouse CTL epitopes were predicted and screened against three mouse MHC-I alleles (H2-Db, H2-Dd, and H2-Kb) (Supporting Information 1: Table S1). Most human HTL epitopes (*n* = 20) exhibited antigenicity scores > 0.45 and were nonallergenic and nontoxic, comprising “MQSSFVADRKKYYDG” (antigenicity: 0.5703), “QKKIRILEPDTPLEK” (antigenicity: 0.7320), “VRTRVVTKKRAKVHP” (antigenicity: 0.9643), “KKIRILEPDTPLEKA” (antigenicity: 0.6029), and “KIRILEPDTPLEKAG” (antigenicity: 0.6061). Notably, IFNepitope predicted 10 HTL peptides could induce IFN-*γ*, while IL4pred predicted 18 could induce IL-4 (Table 4). For mouse HTL epitopes, predictions indicated 23 potential IL-4 inducers and 7 IFN-*γ* inducers. Seven peptides were predicted to induce both cytokines (Supporting Information 2: Table S2).

### 3.6. Immune Simulation Profile

**Figure 6:**
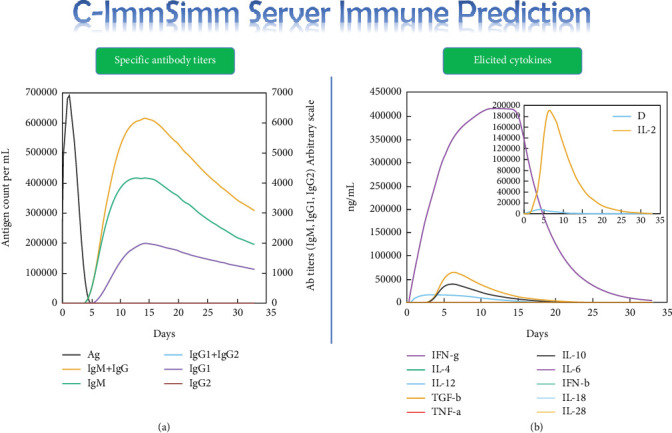
Titers of elicited specific (a) IgG and IgM antibodies and (b) cytokines in response to the administration of SABP1 as a vaccine candidate. Prediction was done using the C-ImmSim web server.

The prediction was done in a 35-day time frame using the C-ImmSim web server. The highest IgG + IgM responses reached about 6000 during 12 days postinoculation (pi), among which the highest IgG1 and IgM titers alone were over 1500 (in 15 days pi) and 4000 (in 12 days pi), respectively (Figure 6a). The highest peaks of the IFN-*γ* were over 400000 ng/mL, showing a strong stimulation of the Th1 immune responses required for the clearance of the invasive free or intracellular tachyzoites (Figure 6b).

## 4. Discussion

The prevalence of *T. gondii* infection is a worldwide health issue. Many prevention methods can be implemented, but basically vaccination is the most effective mode of preventing toxoplasmosis. During the early 2000s, a MEV, MenB, for use against meningococcal B, was developed using structure-based design. The successful development and approval of MenB opened the door for multiple MEV projects against cancer and diseases caused by protozoan pathogens. *T. gondii* SABP1 protein is a good candidate for vaccine development because of its role in parasite adherence and invasion to host cells. In this study, we used bioinformatics methods to characterize specific features of SABP1 to identify immunodominant fragments.

The protein has a MW of 33.73 kDa. As effective immunogens typically exceed 5–10 kDa, SABP1 is a promising immunogenic candidate. Although predicted to be unstable in silico, SABP1 demonstrated favorable thermotolerance (high aliphatic index) and hydrophilicity (low GRAVY score). SABP1 is nonallergenic and antigenic, two essential characteristics for an effective vaccine candidate. While the critical role of PTMs in cellular regulation is well-established, we characterized PTMs specific to SABP1 [38]. We employed the NetPhos 3.1, NetOGlyc 4.0, and NetNGlyc 1.0 as well as the GPS-PAIL servers to predict the phosphorylation, O- and N-glycosylation sites, and lysine acetylation regions for the SABP1 protein, respectively. SABP1 contained 25 predicted phosphorylation sites and 15 O-glycosylation sites. Including other modifications (e.g., N-glycosylation, acetylation), a total of 46 PTM sites were predicted. DeepLoc predicted the cytoplasm as the most likely subcellular localization for SABP1. The protein lacked putative signal peptides and transmembrane domains. These in silico predictions (MW, stability, solubility, localization, and absence of signal peptides/transmembrane domains) provide crucial information for selecting appropriate expression systems and purification strategies. NetSurfP-3.0 predicted random coils and extended strands as the predominant secondary structures in SABP1. Stable secondary structures like alpha-helices and beta-sheets, often stabilized by high hydrogen bond energies, can contribute to structural integrity and potentially form strong epitopes for antibody binding. The presence of these stable elements in SABP1 supports its potential to elicit a strong antibody response [39]. As protein function is intrinsically linked to tertiary structure, elucidating the 3D conformation of SABP1 is essential [39]. The powerful Robetta server was utilized for 3D homology modeling of proteins. Accordingly, we predicted the 3D structure of SABP1, with a C-score of 0.60, showing good homology modeling confidence for this macromolecule.

*T. gondii* infection induces robust cell-mediated and humoral immune responses [40, 41]. Specific IgG antibodies block parasite attachment to host cells. Furthermore, antibodies promote rapid clearance of *T. gondii* via opsonization, enabling phagocytosis by immune cells like macrophages [41]. Conversely, T cell-derived IFN-*γ* is critical for limiting both acute and chronic infection. This important cytokine halts the reactivation of the tissue cysts. While both CD_4_^+^ and CD_8_^+^ T cells contribute to infection control, CD_8_^+^ T cells and their production of IFN-*γ* play a dominant role [40–42]. Analyzing pathogen protein epitopes elucidates virulence and immune evasion mechanisms, guiding epitope-based vaccine design via reverse vaccinology [43, 44]. Epitope prediction relies heavily on protein structural features. Since no single parameter provides comprehensive epitope characterization, multiple indices—including antigenicity, allergenicity, and solubility—are essential for accurate prediction [45–49]. Peptides meeting thresholds for key indices are typically prioritized as candidate epitopes. To enhance prediction robustness given differing algorithm methodologies, we employed three servers for linear B-cell epitope prediction: ABCpred, SVMTriP, and the ElliPro linear tool. Epitopes identified by at least two servers were subsequently screened based on antigenicity, allergenicity, and solubility thresholds. Screening identified four high-potential linear B-cell epitopes from the initial 20 candidates based on antigenicity, non-allergenicity, and solubility, comprising “PGKRIDEEELVPDS” (antigenicity: 1.0986), “GLEKDMQSSFVADRK” (antigenicity: 0.6031), “TREAAADGPTVRTRVV” (antigenicity: 0.7968), and “EELVPDS” (antigenicity: 1.4457). Conformational B-cell epitope prediction using ElliPro identified five significant regions likely involved in antigen-antibody binding, potentially enhancing antibody-mediated immunity.

T-cell-mediated immunity is critical for controlling *T. gondii* infection, as the parasite is obligate intracellular [40]. Consequently, defining the specific T-cell response profile is essential for developing effective vaccines [40]. We used the IEDB server to predict IC50 values for peptides binding to both MHC-I and MHC-II molecules. Lower IC50 values (or percentile ranks) indicate higher binding affinity and stronger potential T-cell epitopes. Predictions revealed potent CTL epitopes binding human and mouse MHC class I. Eight human CTL epitopes demonstrated high immunogenicity scores using Class I immunogenicity prediction of the IEDB server, encompassing “KYYDGWATF,” “KKYYDGWATF,” “AVVPDTFVK,” “EVSEFVAFA,” “TVRTRVVTK,” “SLISFVPAK,” “EAAADGPTVR,” and “LSGPGVLAY.” In addition, several human HTL epitopes in SABP1 of *T. gondii* could elicit both examined cytokines (IFN-*γ* and IL-4), including “QSSFVADRKKYYDGW,” “RTRVVTKKRAKVHPK,” “QKKIRILEPDTPLEK,” “VRTRVVTKKRAKVHP,” “DMQSSFVADRKKYYD,” “SQKKIRILEPDTPLE,” “KKIRILEPDTPLEKA,” “YSQKKIRILEPDTPL,” and “KIRILEPDTPLEKAG.” Notably, several CTL and HTL epitopes also showed strong binding and cytokine induction potential for selected mouse MHC alleles. C-ImmSim simulations predicted robust stimulation of both humoral and cell-mediated immune responses following vaccination. This study focused on in silico prediction of immunodominant regions within SABP1 suitable for MEV design.

The study was conducted exclusively in silico, providing some foundations for the research questions; however, wet lab experiments remain the most important part of a vaccine design study. The primary limitations of our study were as follows: (1) absence of wet lab experiments: The antigenicity, allergenicity, solubility, subcellular localization, and especially the immunogenicity of the identified B-cell and T-cell epitopes have not been confirmed using an immunological assay (e.g., ELISA, cell proliferation assay) or in vivo studies; (2) modeling limitations: The predicted tertiary structure (Robetta) is only as accurate as the templates and algorithms available, which may not accurately model the native protein conformation; and (3) prediction tool reliance: The results reported here are based on the thresholds and algorithms run by the specific bioinformatics tools used in the current study, causing server-dependent bias. The selection of different web tools, algorithms, or parameters would likely yield different results.

## 5. Conclusions

Our extensive in silico data analysis characterized the *T. gondii* SABP1 protein, showing promising characteristics relevant to vaccine development. It was determined that SABP1 is predicted to be a soluble, hydrophilic, nonallergenic, and antigenic cytoplasmic protein. The computational epitope mapping also revealed several potential B cell and T cell immunogenic epitopes, which could be used to develop effective vaccine candidates using a rational design of multi-epitope toxoplasmosis vaccines. The findings provide a valuable foundation and specific leads for future research. However, the immunogenicity and protective efficacy of these predicted epitopes must be rigorously confirmed through experimental validation in immunological assays and animal models.

## Figures and Tables

**Table 1:** Web links to all bioinformatics online servers used in the present study.

**Online servers**	**Predictions**	**Web address**
ABCpred	Linear B-cell epitopes	http://crdd.osdd.net/raghava/abcpred/
AllergenFP v1.0	Allergenicity	https://ddg-pharmfac.net/AllergenFP/
AllerTOP v2.0	Allergenicity	https://www.ddg-pharmfac.net/AllerTOP/
C-ImmSim	Immune simulation	https://kraken.iac.rm.cnr.it/C-IMMSIM/index.php
DeepLoc 2.0	Subcellular localization	https://services.healthtech.dtu.dk/services/DeepLoc-2.0/
DeepTMHMM 2.0	Transmembrane domains	https://dtu.biolib.com/DeepTMHMM
ElliPro	Linear and continuous B-cell epitopes	http://tools.iedb.org/ellipro/
Expasy ProtParam	Physicochemical properties	https://web.expasy.org/protparam/
GPSPAIL	Acetylation on internal lysines	http://pail.biocuckoo.org/
GalaxyRefine	3D refinement	https://galaxy.seoklab.org/cgi-bin/submit.cgi?type=REFINE
IEDB class I Immunogenicity	Immunogenicity	http://tools.iedb.org/immunogenicity/
IEDB MHCI	MHC-I binders	http://tools.iedb.org/mhci/
IEDB MHCII	MHC-II binders	http://tools.iedb.org/mhcii/
IFNepitope	IFN-*γ* induction	http://crdd.osdd.net/raghava/ifnepitope/
IL4pred	IL-4 induction	https://webs.iiitd.edu.in/raghava/il4pred/predict.php
PROCHECK	3D validation	https://saves.mbi.ucla.edu/
NetSurfP-3.0	Secondary structure	https://services.healthtech.dtu.dk/services/NetSurfP-3.0/
NetNGlyc 1.0	N-glycosylation sites	http://www.cbs.dtu.dk/services/NetNGlyc/
NetOGlyc 4.0	O-glycosylation sites	https://services.healthtech.dtu.dk/services/NetOGlyc-4.0/
NetPhos 3.1	Phosphorylation sites	https://services.healthtech.dtu.dk/services/NetPhos-3.1/
PepCalc	Peptide solubility	https://pepcalc.com/peptide-solubility-calculator.php
Protein-Sol	Protein solubility	https://protein-sol.manchester.ac.uk/
Robetta	tertiary structure	https://robetta.bakerlab.org/
SignalP-6.0	Signal peptide	https://services.healthtech.dtu.dk/services/SignalP-6.0/
SVMTriP	Linear B-cell epitopes	http://sysbio.unl.edu/SVMTriP/
ToxinPred	Toxicity	https://webs.iiitd.edu.in/raghava/toxinpred/algo.php
ToxoDB	Sequence retrieval	https://toxodb.org/toxo/app
VaxiJen v. 2.0	Antigenicity	http://www.ddg-pharmfac.net/vaxijen/VaxiJen/VaxiJen.html

**Table 2:** Shared continuous B-cell epitopes predicted for the *T. gondii* SABP1 using three different web servers (ABCpred, SVMTriP, and ElliPro) and screened in terms of antigenicity, allergenicity, and solubility.

**Epitope no.**	**Epitope sequence**	**Antigenicity (VaxiJen server)**	**Allergenicity (AllergenFP server)**	**Solubility (PepCalc server)**
1	KIRILEPDTPLEKAGV	0.4170	Yes	Good
2	SAAGTPPPPPEACTG	0.2604	Yes	Good
3	NNPSPGYSDSGCYY	0.2512	No	Poor
4	CRPAPPGKRIDE	1.2425	Yes	Good
5	SNTPL	0.456	Yes	Good
6	PGYSDSGCYY	0.3388	Yes	Poor
7	GVEIEEGETAPSSAGA	0.6430	Yes	Good
8	DVPTMEVSEFVAFAN	0.2998	No	Poor
9	PTAPLGM	0.2145	Yes	Poor
10	TAPLGMGVYLVY	0.5442	Yes	Poor
11	SFVADRKK	0.6753	Yes	Good
12	EAAADGPTVRTRVVTK	1.0328	Yes	Good
13	VPDSTTTEEALKE	0.3321	No	Good
14	PGKRIDEEELVPDS^a^	1.0986	No	Good
15	EPKKEVGA	0.8901	Yes	Good
16	WATFLKQMDAADGT	0.2354	No	Good
17	GLEKDMQSSFVADRK^a^	0.6031	No	Good
18	DGVEIEEGETAPSSAG	0.4991	Yes	Good
19	TREAAADGPTVRTRVV^a^	0.7968	No	Good
20	EELVPDS^a^	1.4457	No	Good

^a^Potentially qualified epitopes.

**Table 3:** Human cytotoxic T-lymphocyte (CTL) specific epitope prediction for *T. gondii* SABP1 proteins against IEDB HLA reference set and subsequent screening regarding immunogenicity, allergenicity, and toxicity.

**Protein**	**Alleles from HLA reference set**	**Start-end**	**CTL epitope**	**Percentile rank**	**Immunogenicity**	**Allergenicity**	**Toxicity**
*T. gondii* SABP1	HLA-A∗23:01	57-65	KYYDGWATF	0.01	0.31833	Yes	No
	HLA-A∗24:02	57-65	KYYDGWATF	0.01	0.31833	Yes	No
	HLA-B∗07:02	23-31	RPAPPGKRI	0.02	−0.00751	Yes	No
	HLA-A∗30:01	22-30	KVHPKSAVK	0.01	−0.10886	Yes	No
	HLA-A∗24:02	56-65	KKYYDGWATF	0.01	0.31389	No	No
	HLA-A∗23:01	56-65	KKYYDGWATF	0.01	0.31389	No	No
	HLA-A∗03:01	22-30	KVHPKSAVK	0.01	−0.12912	Yes	No
	HLA-B∗35:01	49-57	VPTMEVSEF	0.02	−0.30925	Yes	No
	HLA-A∗11:01	38-46	AVVPDTFVK	0.01	0.1833	Yes	No
	HLA-A∗68:01	24-32	EVGAFKFQK	0.05	−0.30925	Yes	No
	HLA-A∗68:02	60-69	EPTAPLGMGV	0.02	−0.06613	No	No
	HLA-B∗07:02	8-16	TPLSGPGVL	0.04	−0.09479	Yes	No
	HLA-A∗68:02	53-61	EVSEFVAFA	0.02	0.30133	Yes	No
	HLA-A∗68:02	70-78	ETAPSSAGA	0.02	−0.13764	Yes	No
	HLA-A∗30:01	10-18	TVRTRVVTK	0.01	0.20264	No	No
	HLA-A∗03:01	66-74	SLISFVPAK	0.03	0.04309	No	No
	HLA-A∗11:01	1-9	ATTLQPPPK	0.02	−0.22788	No	No
	HLA-A∗03:01	3-11	TLQPPPKVK	0.03	−0.26247	No	No
	HLA-A∗68:01	3-12	EAAADGPTVR	0.09	0.15311	No	No
	HLA-A∗01:01	10-18	LSGPGVLAY	0.04	0.0852	Yes	No

**Table 4:** Human helper T-lymphocyte (HTL) specific epitope prediction for *T. gondii* SABP1 against HLA reference set with subsequent screening regarding antigenicity, allergenicity, toxicity, and cytokine (IFN-*γ*, IL-4) induction.

**Protein**	**Alleles from HLA reference set**	**Start–end**	**HTL epitope**	**Percentile rank**	**Antigenicity**	**Allergenicity**	**Toxicity**	**IFN-*γ* induction**	**IL-4 induction**
*T. gondii* SABP1	HLA-DRB1∗13:02	47-61	MQSSFVADRKKYYDG	0.02	0.5703	No	No	Negative	**Positive**
	HLA-DRB1∗13:02	48-62	QSSFVADRKKYYDGW	0.03	0.3338	No	No	**Positive**	**Positive**
	HLA-DRB1∗13:02	12-26	RTRVVTKKRAKVHPK	0.06	1.2087	Yes	No	**Positive**	**Positive**
	HLA-DRB4∗01:01	17-31	QKKIRILEPDTPLEK	0.09	0.7320	No	No	**Positive**	**Positive**
	HLA-DQA1∗05:01/DQB1∗03:01	63-77	GVEIEEGETAPSSAG	0.12	0.6633	Yes	No	Negative	**Positive**
	HLA-DRB1∗13:02	11-25	VRTRVVTKKRAKVHP	0.14	0.9643	No	No	**Positive**	**Positive**
	HLA-DRB1∗13:02	46-60	DMQSSFVADRKKYYD	0.14	0.3350	No	No	**Positive**	**Positive**
	HLA-DRB4∗01:01	16-30	SQKKIRILEPDTPLE	0.14	0.8054	Yes	No	**Positive**	**Positive**
	HLA-DQA1∗05:01/DQB1∗02:01	38-52	PDSTTTEEALKEVDE	0.17	0.0425	No	No	Negative	Negative
	HLA-DQA1∗05:01/DQB1∗03:01	62-76	DGVEIEEGETAPSSA	0.19	0.4348	No	No	Negative	**Positive**
	HLA-DRB1∗04:01	53-67	EVSEFVAFANNVGSL	0.2	0.1420	No	No	Negative	**Positive**
	HLA-DQA1∗05:01/DQB1∗02:01	37-51	VPDSTTTEEALKEVD	0.25	0.0305	No	No	**Positive**	Negative
	HLA-DRB1∗04:01	52-66	MEVSEFVAFANNVGS	0.25	0.2844	No	No	Negative	**Positive**
	HLA-DRB1∗04:01	54-68	VSEFVAFANNVGSLI	0.26	0.4449	Yes	No	Negative	**Positive**
	HLA-DRB4∗01:01	18-32	KKIRILEPDTPLEKA	0.29	0.6029	No	No	**Positive**	**Positive**
	HLA-DRB1∗03:01	20-34	IRILEPDTPLEKAGV	0.32	0.2715	No	No	Negative	**Positive**
	HLA-DRB4∗01:01	15-29	YSQKKIRILEPDTPL	0.35	0.9266	Yes	No	**Positive**	**Positive**
	HLA-DRB1∗03:01	19-33	KIRILEPDTPLEKAG	0.35	0.6061	No	No	**Positive**	**Positive**
	HLA-DRB1∗03:01	47-61	MQSSFVADRKKYYDG	0.39	0.5703	No	No	Negative	**Positive**
	HLA-DQA1∗05:01/DQB1∗03:01	61-75	EDGVEIEEGETAPSS	0.41	0.5872	Yes	No	Negative	**Positive**

Positive cytokine (IFN-gamma, IL4) induction by each epitope is shown in bold.

## Data Availability

The data that support the findings of this study are available from the corresponding author upon reasonable request.

## Supporting Information

Supporting Information 1 Table S1: Mouse cytotoxic T-lymphocyte (CTL) specific epitope prediction for *T. gondii* SABP1 proteins against some mouse MHC-I alleles and subsequent screening regarding immunogenicity, allergenicity, and toxicity.

Supporting Information 2 Table S2: Mouse helper T-lymphocyte (HTL) specific epitope prediction for *T. gondii* SABP1 proteins against some mouse MHC-II alleles and subsequent screening regarding antigenicity, allergenicity, toxicity, and cytokine (IFN-*γ* and IL-4) induction.
