# Supplementary material for: Sialic Acid–Binding Protein-1 (SABP1) of Toxoplasma gondii: Preliminary Computer-Based Epitope Mapping for Enhanced Vaccine Design
Source: J Parasitol Res. 2025 Sep 3;2025:9909421. doi: 10.1155/japr/9909421 (PMC12422853; doi:10.1155/japr/9909421)
Supplement: Supporting Information 1 — Table S1: Mouse cytotoxic T-lymphocyte (CTL) specific epitope prediction for T. gondii SABP1 proteins against some mouse MHC-I alleles and subsequent screening regarding immunogenicity, allergenicity, and toxicity. [file 9909421.f1.docx]

**Supplementary Table 1.** Mouse cytotoxic T-lymphocyte (CTL) specific epitope prediction for *T. gondii* SABP1 proteins against some mouse MHC-I alleles and subsequent screening regarding immunogenicity, allergenicity, and toxicity.

| Protein | Mouse MHC allele | Start-End | CTL epitope | Percentile rank | Immunogenicity | Allergenicity | Toxicity |
| --- | --- | --- | --- | --- | --- | --- | --- |
| *T. gondii*  SABP1 | H2-Db | 55-66 | FGEEAVEDGVEI | 1.8 | 0.45589 | Yes | No |
|  |  | 67-78 | KQMDAADGTLVL | 3.6 | 0.14742 | No | No |
|  |  | 1-12 | ATTLQPPPKVKV | 4.0 | -0.05425 | No | No |
|  |  | 39-50 | SAAGTPPPPPEA | 4.6 | 0.0909 | Yes | No |
|  |  | 4-15 | AAADGPTVRTRV | 7.1 | 0.24694 | No | No |
|  |  | 8-19 | TPLSGPGVLAYI | 7.2 | -0.31635 | No | No |
|  |  | 7-18 | SPGYSDSGCYYL | 7.6 | -0.43792 | Yes | No |
|  |  | 25-36 | APPGKRIDEEEL | 7.7 | 0.25935 | No | No |
|  |  | 28-39 | FKFQKKDGVQHI | 8.3 | -0.48396 | No | No |
|  |  | 36-47 | LVPDSTTTEEAL | 8.3 | 0.17263 | No | No |
|  | H2-Dd | 1-12 | ATTLQPPPKVKV | 2.3 | -0.43792 | No | No |
|  |  | 8-19 | TPLSGPGVLAYI | 3.3 | -0.05425 | No | No |
|  |  | 36-47 | LVPDSTTTEEAL | 3.9 | 0.17263 | No | No |
|  |  | 25-36 | APPGKRIDEEEL | 4.4 | 0.25935 | No | No |
|  |  | 36-47 | LMAVVPDTFVKF | 4.6 | 0.16208 | No | No |
|  |  | 39-50 | SAAGTPPPPPEA | 4.9 | 0.0909 | Yes | No |
|  |  | 67-78 | KQMDAADGTLVL | 6.6 | 0.14742 | No | No |
|  |  | 4-15 | AAADGPTVRTRV | 6.7 | 0.24694 | No | No |
|  |  | 12-23 | VAVYSQKKIRIL | 9.7 | -0.4375 | No | No |
|  |  | 4-15 | LQPPPKVKVAVY | 9.9 | -0.30936 | Yes | No |
|  | H2-Kb | 36-47 | LMAVVPDTFVKF | 7.7 | 0.16208 | No | No |
|  |  | 17-28 | AYIEPKKEVGAF | 12 | -0.09903 | Yes | No |
|  |  | 36-47 | LVPDSTTTEEAL | 12 | 0.17263 | No | No |
|  |  | 39-50 | SAAGTPPPPPEA | 12 | 0.0909 | Yes | No |
|  |  | 25-36 | VGAFKFQKKDGV | 13 | -0.43792 | No | No |
|  |  | 54-65 | DRKKYYDGWATF | 13 | 0.02461 | No | No |
|  |  | 39-50 | VVPDTFVKFDVP | 13 | 0.15376 | No | No |
|  |  | 1-12 | ATTLQPPPKVKV | 14 | -0.45278 | No | No |
|  |  | 58-69 | YYDGWATFLKQM | 14 | 0.18678 | Yes | No |
|  |  | 18-29 | KKIRILEPDTPL | 14 | 0.34794 | No | No |
