# Supplementary material for: Sialic Acid–Binding Protein-1 (SABP1) of Toxoplasma gondii: Preliminary Computer-Based Epitope Mapping for Enhanced Vaccine Design
Source: J Parasitol Res. 2025 Sep 3;2025:9909421. doi: 10.1155/japr/9909421 (PMC12422853; doi:10.1155/japr/9909421)
Supplement: Supporting Information 2 — Table S2: Mouse helper T-lymphocyte (HTL) specific epitope prediction for T. gondii SABP1 proteins against some mouse MHC-II alleles and subsequent screening regarding antigenicity, allergenicity, toxicity, and cytokine (IFN-γ and IL-4) induction. [file 9909421.f2.docx]

**Supplementary Table 2.** Mouse helper T-lymphocyte (HTL) specific epitope prediction for *T. gondii* SABP1 proteins against some mouse MHC-II alleles and subsequent screening regarding antigenicity, allergenicity, toxicity, and cytokine (IFN-γ, IL-4) induction.

| Protein | Mouse MHC allele | Start-End | HTL epitope | Percentile rank | Antigenicity | Allergenicity | Toxicity | IFN-γ induction | IL-4 induction |
| --- | --- | --- | --- | --- | --- | --- | --- | --- | --- |
| *T. gondii*  SABP1 | H2-IAb | 66-80 | IEEGETAPSSAGAEV | 0.30 | 0.7297 | No | No | Negative | **Positive** |
|  |  | 65-79 | EIEEGETAPSSAGAE | 0.44 | 0.6086 | No | No | Negative | **Positive** |
|  |  | 33-47 | PKLFTGSAAGTPPPP | 0.47 | 0.8524 | No | No | Negative | Negative |
|  |  | 32-46 | QPKLFTGSAAGTPPP | 0.76 | 1.1053 | Yes | No | Negative | Negative |
|  |  | 47-61 | PPEACTGPLPEPAEP | 1.40 | 0.5153 | Yes | No | Negative | Negative |
|  |  | 48-62 | PEACTGPLPEPAEPT | 1.40 | 0.7441 | No | No | Negative | Negative |
|  |  | 31-45 | SQPKLFTGSAAGTPP | 1.70 | 0.8288 | No | No | Negative | Negative |
|  |  | 64-78 | VEIEEGETAPSSAGA | 1.90 | 0.5375 | No | No | Negative | **Positive** |
|  |  | 34-48 | KLFTGSAAGTPPPPP | 2 | 0.8657 | Yes | No | Negative | Negative |
|  |  | 46-60 | PPPEACTGPLPEPAE | 2.40 | 0.3821 | No | No | Negative | Negative |
|  | H2-IAd | 58-72 | EAVEDGVEIEEGETA | 0.74 | 0.4994 | No | No | **Positive** | **Positive** |
|  |  | 57-71 | EEAVEDGVEIEEGET | 1.20 | 0.5089 | No | No | **Positive** | **Positive** |
|  |  | 64-78 | VEIEEGETAPSSAGA | 2.20 | 0.5375 | No | No | Negative | **Positive** |
|  |  | 56-70 | GEEAVEDGVEIEEGE | 2.60 | 0.6252 | Yes | No | **Positive** | **Positive** |
|  |  | 65-79 | EIEEGETAPSSAGAE | 2.80 | 0.6086 | No | No | Negative | **Positive** |
|  |  | 63-77 | GVEIEEGETAPSSAG | 2.80 | 0.6633 | Yes | No | Negative | **Positive** |
|  |  | 59-73 | AVEDGVEIEEGETAP | 3.40 | 0.2444 | No | No | **Positive** | **Positive** |
|  |  | 13-27 | PGVLAYIEPKKEVGA | 4.20 | 0.6452 | No | No | Negative | **Positive** |
|  |  | 12-26 | GPGVLAYIEPKKEVG | 5.10 | 0.4185 | Yes | No | Negative | **Positive** |
|  |  | 62-76 | DGVEIEEGETAPSSA | 5.20 | 0.4348 | No | No | Negative | **Positive** |
|  | H2-IEd | 20-34 | RAKVHPKSAVKSQPK | 0.41 | 1.0630 | No | No | Negative | **Positive** |
|  |  | 8-22 | GPTVRTRVVTKKRAK | 0.60 | 1.0886 | No | No | **Positive** | **Positive** |
|  |  | 19-33 | KRAKVHPKSAVKSQP | 0.84 | 0.8408 | No | No | Negative | **Positive** |
|  |  | 12-26 | RTRVVTKKRAKVHPK | 1.20 | 1.2087 | Yes | No | **Positive** | **Positive** |
|  |  | 7-21 | DGPTVRTRVVTKKRA | 1.20 | 0.5822 | No | No | Negative | **Positive** |
|  |  | 25-39 | VGAFKFQKKDGVQHI | 1.30 | -0.8113 | No | No | Negative | **Positive** |
|  |  | 47-61 | MQSSFVADRKKYYDG | 1.30 | 0.5703 | No | No | Negative | **Positive** |
|  |  | 48-62 | QSSFVADRKKYYDGW | 1.60 | 0.3338 | No | No | **Positive** | **Positive** |
|  |  | 24-38 | EVGAFKFQKKDGVQH | 1.60 | -0.5838 | No | No | Negative | **Positive** |
|  |  | 12-26 | VAVYSQKKIRILEPD | 1.90 | 0.8552 | No | No | Negative | **Positive** |
